# Syphilis Among U.S.-Bound Refugees, 2015 – 2018

**DOI:** 10.1007/s10903-024-01609-2

**Published:** 2024-06-07

**Authors:** Shannon Fox, Priti Shah, Michelle Russell Hollberg, Deborah Lee, Drew L. Posey

**Affiliations:** 1 Division of Global Migration Health, Centers for Disease Control and Prevention, Atlanta, GA, USA; 2 ORISE Research Participant, Division of Global Migration Health, Centers for Disease Control and Prevention, Atlanta, GA, USA

**Keywords:** Refugees, Syphilis, Screening, Sexually transmitted diseases

## Abstract

We assessed syphilis screening data from overseas medical examinations among U.S.-bound refugees to characterize seropositive syphilis cases and treatment from January 1, 2015, to December 31, 2018. During this time period, all refugees 15 years and older were required to undergo syphilis screening prior to resettlement to the United States. Of the 160,381 refugee arrivals who had a syphilis screening performed, 697 (434 per 100,000) were diagnosed with any stage (infectious or non-infectious) of syphilis. Among the 697 persons with seropositive syphilis, a majority (63%) were from the Africa region and were male (58%), and 53 (7.6%) were diagnosed with an infectious stage of syphilis. All infectious cases were treated prior to resettlement. This information suggests a comparable risk of infection among U.S.-bound refugees compared to a report of syphilis among U.S.-bound refugees from 2009 to 2013, indicating low rates in this population for at least a decade.

## Background

Syphilis is a curable sexually transmitted infection (STI) caused by the bacterium *Treponema pallidum*. If left untreated, it can lead to long-term organ system damage [1, 2]. The World Health Organization estimated 376 million cases of STIs for 2018 including 6 million (1.6%) syphilis cases [3]. The United States (U.S.) Centers for Disease Control and Prevention (CDC) has regulatory responsibility to require medical examinations for refugee populations before they may be admitted to the U.S. to screen for inadmissible conditions like syphilis [4]. CDC issues Technical Instructions (TIs) for panel physicians (licensed medical doctors who perform the required immigration medical examination overseas) to follow when performing medical examinations on refugee populations [4]. U.S. bound refugees are screened according to the traditional syphilis screening algorithm—which starts with a non-treponemal serologic test that, if reactive, is followed by a confirmatory treponemal serologic test. Confirmed syphilis cases must be treated prior to departure for the U.S [5]. . Prior to 2021, all refugees aged 15 years or older applying for entry into the U.S. were required to complete an overseas medical examination including screening for syphilis. This report is a continuation of a previous analysis of syphilis seropositive rates among U.S.-bound refugees aged 15 years or older from 2009 to 2013 [6]. The former report found that seropositive syphilis among U.S.-bound refugees was found to be 373 cases per 100,000 refugees but was limited in its ability to account for syphilis staging data and infectious syphilis rates [6]. The syphilis staging data are important for understanding the risk of syphilis transmission since, outside of the setting of mother to child transmission during pregnancy, syphilis is only infectious during the primary and secondary stages and is not considered infectious during latent or tertiary stages [1, 7]. Therefore, the stages of syphilis—primary, secondary, early/late latent, and tertiary—were added to the overseas medical examinations during 2014. Congenital and neurosyphilis were also added due to their ability to occur during any stage of syphilis [1]. This report aims to be the first to describe overall syphilis seropositivity, staging, and treatment among refugees who resettled to the U.S. from 2015 to 2018. Results of this report can be utilized to better understand burden and transmission risk of syphilis in this population.

## Methods

Data for overseas medical examinations are captured in the CDC’s Electronic Disease Notification System (EDN), a centralized electronic reporting system [7]. We analyzed EDN data on all refugees 15 years or older at the time of exam who arrived in the U.S. during January 1, 2015, to December 31, 2018. These data were linked to the U.S. Department of State’s (DOS) Worldwide Refugee Admission Processing System (WRAPS) to gather additional data not captured in EDN, including nationality and pre-resettlement location.

A seropositive syphilis case was defined as having a positive screening test [(Venereal Disease Research Laboratory (VDRL), Rapid Plasma Reagin (RPR), or unspecified screening test)] and a positive confirmatory test [(*T. pallidum* passive particle agglutination assay (TP-PA), *Treponema pallidum* hemagglutination test (TPHA), *Treponema pallidum* enzyme immunoassay (EIA) or chemiluminescence immunoassay (CIA), or immunoblots, fluorescent treponemal antibody absorbed test (FTA-ABS), rapid treponemal assay, or unspecified confirmatory test)], irrespective of the order in which the tests were performed [5]. Demographic characteristics included sex, age group, region of origin based on nationality, and pre-settlement location. Pregnancy status was also reviewed due to concern of the rising incidence of congenital syphilis in the U.S [8]. . Age groups at the time of exam were categorized as: 15–24, 25–44, 45–64, and 65 + years. Region of origin was based on the Department of States (DOS) classifications as Africa, East Asia/Pacific, Europe/Eurasia, Near East, South/Central Asia, and Western Hemisphere [9]. Pre-settlement location was defined as having been in a refugee camp versus a non-camp setting.

A person with seropositive syphilis was considered treated if the examination record reported current or previous syphilis treatment or treatment dates. Syphilis staging was reported as ‘infectious’ or ‘non-infectious’ and divided into different stages: infectious including primary and secondary; non-infectious including latent and tertiary stages [1]. Syphilis staging also captured congenital and neuro-syphilis. The CDC Technical Instructions stage syphilis according to CDC guidelines [10].

All analyses were performed using SAS 9.4. The proportion of refugees with syphilis screening was determined by dividing the number of refugees with at least one syphilis test result by the total number of refugees examined in EDN. Proportion of seropositive syphilis cases was determined by dividing the number of refugees with both screening and confirmatory seropositivity by the total number of refugees screened for syphilis. Adjusted odds ratios were calculated to determine the associations between a positive screening result, sex, age group, region, and pre-settlement location. Age-based infectious syphilis rates were calculated and compared to 2018 U.S. primary and secondary syphilis rates.

This assessment was reviewed in accordance with CDC institutional review policies and procedures and was determined to be non-research program evaluation.

## Results

A total of 173,243 refugees 15 years and older arrived in the U.S. between January 1, 2015, and December 31, 2018, and had data recorded in the EDN system. Of these arrivals, 160,381 (93%) were screened for syphilis, 697 (< 1%) were seropositive, and 53 (0.03%) were classified as infectious. The syphilis prevalence rate for U.S.-bound refugees was 434 cases per 100,000 refugees and the prevalence rate of infectious syphilis was 33 cases per 100,000 refugees (Tables 1 and 2). The age of refugees with seropositive syphilis ranged from 15 to 91 years (median: 42, interquartile range: 22). The age of refugees with infectious syphilis ranged from 18 to 82 years (median: 37, interquartile range: 16). It is important to note that 70% (37 cases) of infectious cases were in refugees between the ages of 18–44, whereas only 30% (16 cases) were in those 45 + years old. A total of 406 (58%) seropositive cases occurred among males and 429 (61%) occurred in non-camp settings. Syphilis seroprevalence was highest among refugees from the Africa region (915 cases per 100,000) and the highest number of cases was in refugees from the Democratic Republic of the Congo (Table 1).

Among 85,514 female records in EDN, there were 2,439 pregnant applicants at the time of exam and 2,424 (> 99%) were screened for syphilis. Among the 697 seropositive syphilis cases, 6 (< 1%) were pregnant at the time of exam, all were listed as non-infectious syphilis, and all were treated prior to arrival. The 6 pregnant cases were all in their first or second trimester, and all were treated with benzathine penicillin G prior to 30 days before their estimated due date.

A total of 693 (99%) refugees with seropositive syphilis were reported treated, including all those with infectious syphilis (53 cases). The other 4 refugees (1%) were missing treatment data in EDN (Table 2); of these, 3 were listed as having falsely reactive results, and 1 was listed as non-infectious. Of those who were treated, 622 (89%) were treated with benzathine penicillin G and 39 (6%) were treated with doxycycline (Table 2).

Among the 697 seropositive refugees, 419 (60%) were non-infectious, 53 (7.6%) were infectious, and 225 (32%) were reported as unknown (224 with no staging data provided) or other (1 case of non-infectious neurosyphilis) (Table 2). Of those with missing staging data, 204 (91%) had been previously treated for syphilis within the past year with no staging being documented in the exam notes; the other 20 (9%) did not have any information regarding staging or previous syphilis infection listed in the exam notes. Among the 20 seropositive refugees with missing staging data, 17 arrived in 2015 soon after the reporting of staging data was implemented in the Technical Instructions.

The association was assessed between seropositivity and sex, age group, region, and pre-settlement location (Table 1). Female refugees were less likely to be seropositive for syphilis when compared to male. When compared to refugees in the 25–44 age group, those who were 15–24 years old were less likely to be seropositive for syphilis and those who were 45–65 years or older were more likely to be seropositive for syphilis. Compared to those from the Africa region, refugees from the East Asia/Pacific, Near East, South/Central Asia, and Europe/Eurasia regions were less likely to be seropositive for syphilis. In addition, refugees from the Western Hemisphere showed no significant association with being seropositive for syphilis. Refugees in camps were less likely to have syphilis seropositivity when compared to those in non-camp settings.

The infectious syphilis rate among refugees aged 15–24 was 13.8 per 100,000 and was comparable to the rates of primary and secondary syphilis among U.S. populations of the same age which ranged from 7.7 to 28.1 during 2018 [11]. Refugees aged 25–44 had a rate of 37.6 which was only slightly higher than the U.S. rates for the same age group ranging from 13.6 to 32.7. Refugees aged 45–64 had a higher rate of 52.6 compared to the U.S. rates for the same age group ranging from 4.6 to 10.6, and refugees aged 65 + had a much higher rate of 59.6 compared to the U.S. rates for the same age group of 0.8 [11].

## Discussion

The overall syphilis seroprevalence among this refugee population was 434.6 per 100,000, suggesting a comparable risk of transmission among U.S.-bound refugees with the previous report [6]. Infectious syphilis prevalence was found to be 33.0 per 100,000, but earlier reports did not include infectious rates since reporting of staging data was not required prior to 2014 [7]. As for the overall syphilis prevalence rate, the previous report similarly found that overall prevalence among refugees was 373 cases per 100,000 and syphilis seropositivity was associated with the Africa region of origin, male sex, increasing age, and living in non-camp settings [6]. Due to the rising incidence of congenital syphilis transmission in the U.S., pregnancy status was reviewed for female applicants, and 6 (< 1%) were seropositive for syphilis [8]. All pregnant cases were non-infectious and treated with benzathine penicillin G prior to 30 days before their due date, greatly reducing the risk of mother-to-infant transmission [8, 12].

Prevalence patterns in the analysis reflect global syphilis patterns reported by the World Health Organization (WHO) based on regions. This analysis estimated prevalence among refugees from the Africa region and the East Asia/Pacific regions at 1% and 0.4%, respectively. As of 2016, WHO estimated syphilis prevalence as 1.6% for the Africa region and 0.2% for the East Asia/Pacific region [13], which is comparable to refugee prevalence rates in this analysis.

This analysis allows comparisons to be made between the U.S.-bound refugee infectious syphilis rates and infectious syphilis rates of the U.S. population. When comparing the rates of infectious syphilis in refugees to 2018 U.S. rates, infectious rates in refugees aged 15–44 years were comparable to the U.S. rates for that age group, and rates of infectious syphilis were higher in refugees aged 45+ [11]. Higher rates among older refugees could be attributed to the undertesting of older U.S. adults who are seen as lower risk for syphilis, whereas all refugees aged 15 or older were tested during the analysis period [7].

There are a few limitations with this analysis. First, we are unable to compare all refugee syphilis rates to U.S. syphilis rates as U.S. rates include ages younger than 15 years old; however, the data do allow for age comparison of infectious rates among refugees and the U.S. population from 2018 [11]. Second, people exposed to *T. pallidum* have reactive treponemal tests for life even after receiving treatment and can have reactive non-treponemal titers after treatment as well, thus leading to potentially higher syphilis positivity in those who are older and may have already recovered from syphilis or experienced other treponemal infections [6, 14]. Third, human immunodeficiency virus (HIV) is not required to be reported, and we are unable to determine if neurosyphilis is under-reported. Also, RPR titers were not consistently reported. Fourth, missing and/or misclassification of data through manual entry of records into EDN may have resulted in data not being appropriately captured. There were 12,862 records missing testing data in EDN. 96% of these missing records were paper records that were manually entered into the system with a majority missing from Western Hemisphere region, particularly Cuba. The proportion with complete results increased over time, with 82% of missing testing records occurring between 2015 and 2016. Extensive data cleaning was performed to ensure accuracy based on scanned record copies and data collection improvements have since been implemented.

In 2021, CDC updated the syphilis screening Technical Instructions to require screening for refugees between the ages of 18–44 years old [5]. This update occurred since a majority (70%) of infectious cases occurred in this 18–44 age range and comparable rates of infectious syphilis were found between U.S.-bound refugees and the 2018 U.S. population for this specific age range. Refugees aged 18–44 years old were found to have a comparable infectious syphilis rate range (13.8–37.6) when compared to the U.S. infectious syphilis rate range for the same age range (7.7–32.7) [11]. Despite the rates among U.S.-bound refugees being higher than the U.S. rates in those 45 and older, there was still a very low yield of 16 cases (30%) of infectious syphilis among the older population of U.S.-bound refugees. In fact, there was an overall low yield of infectious syphilis across all age groups in the screening program: only 53 infectious cases among the 160,381 refugees screened, or a rate of 33 per 100,000 refugees. While costs of tests may vary in each country, requiring overseas screening for those 45 and older may represent a significant cost and effort to find such a small number of cases. Refugees also receive a benefit for a comprehensive medical examination post-arrival and CDC recommends syphilis screening at this encounter, particularly if no overseas results are available on syphilis screening [15]. Therefore, it is likely the small number of infectious syphilis cases in older populations would be captured after arrival.

This analysis improves upon available information about syphilis seropositivity and burden among U.S.-bound refugees by systematic evaluation of centralized data from overseas medical examinations during 2015–2018. However, further stratification based on risk is difficult because it is beyond the scope of a refugee medical exam to determine if an applicant has sexual risk factors for syphilis. Data should be monitored for determination of whether to propose a regulatory change to the syphilis screening requirement of the overseas medical examination.

## Conclusion

The clinical consequences of seropositive syphilis can be severe and recognizing individual cases of syphilis plays a pivotal role in protecting the health of refugees and the U.S. population. According to these data, most seropositive syphilis cases were classified as non-infectious and suggest a comparable risk of infection when compared to the prior analysis conducted on syphilis among U.S.-bound refugees from 2009 to 2013 [6]. This comparable risk of infection also showcases a low rate of seropositive syphilis among U.S.-bound refugees over the course of at least a decade [6]. The analysis for the first time showcases a low rate of infectious syphilis among U.S.-bound refugees and how all infectious cases were treated prior to refugees’ resettlement to the U.S., even more greatly reducing the risk of transmission within the U.S. Improvements to data collection processes and quality have been facilitated and the CDC Technical Instructions for syphilis screening were updated based on analyses that utilized the data from this report during 2021 [5]. Moving forward, similar analyses could be used with other factors to help inform usefulness and impact of overseas syphilis screening among U.S.-bound refugees and other migrant populations.

## Figures and Tables

**Table 1:** Syphilis screening results and associated demographic characteristics among refugees ages ≥ 15 years old who arrived in the United States from January 1, 2015 - December 31, 2018

	Total EDN^a^ Records		Syphilis Test						Adjusted Odds Ratio for Seropositive Syphilis (95% Confidence Interval)
			Total Screened^b^		Seropositive for Syphilis^c^ (Infectious or non-infectious)		Infectious Syphilis		
	*N*	%	*N*	%(row)	*N*	Per 100,000	*N*	Per 100,000	
Total	173,243	100.0	160,381	92.6	697	434.6	53	33.0	
Demographics									
Sex									
Female	85,514	49.4	79,002	92.4	291	368.3	25	31.6	1.0
Male	87,727	50.6	81,378	92.8	406	498.9	28	34.4	1.4 (1.2–1.6)
Age Group									
15–24	53,732	31.0	50,790	94.5	77	151.6	7	13.8	0.3 (0.2–0.4)
25–14	86,109	49.7	79,857	92.7	321	402.0	30	37.6	1.0
45–64	27,860	16.1	24,736	88.8	241	974.3	13	52.6	2.7 (2.3–3.2)
65+	5,334	3.1	5,035	94.4	58	1,151.9	3	59.6	4.0 (3.0-5.4)
Region of Origin^d^									
Africa	48,785	28.2	47,840	98.1	442	923.9	28	58.5	1.0
D.R.C.	23,288	13.4	22,859	98.2	237	1036.8	16	70.0	-
Somalia	12,128	7.0	11,989	98.9	73	608.9	2	16.7	-
Eritrea	4,601	2.7	4,544	98.8	35	770.2	2	44.0	-
Ethiopia	2,591	1.5	2,487	96.0	32	1286.7	4	160.8	-
Sudan	2,283	1.3	2,231	97.7	34	1524.0	2	89.6	-
East Asia/Pacific	25,569	14.8	25,205	98.6	97	384.8	3	11.9	0.3 (0.3–0.4)
Burma	22,656	13.1	22,559	99.6	79	350.2	3	13.3	-
Near East	37,143	21.4	35,647	96.0	72	202.0	2	5.6	0.1 (0.1–0.2)
Iraq	18,781	10.8	17,994	95.8	57	316.8	2	11.1	-
Syria	10,435	6.0	10,246	98.2	6	58.6	0	0.0	-
Iran	7,552	4.4	7,087	93.8	7	98.8	0	0.0	-
South/Central Asia	30,925	17.9	30,174	97.6	51	169.0	15	49.7	0.2 (0.1–0.2)
Afghanistan	17,008	9.8	16,472	96.8	28	170.0	0	0.0	-
Bhutan	10,424	6.0	10,357	99.4	16	154.5	12	115.9	-
Western Hemisphere	20,118	11.6	11,043	54.9	31	280.7	4	36.2	0.7 (0.4–1.2)
Cuba	16,730	9.7	8,011	47.9	15	187.2	2	25.0	-
El Salvador	1,857	1.1	1,624	87.5	5	307.9	2	123.2	-
Europe/Eurasia	10,101	5.8	10,011	99.1	4	40.0	0	0.0	0.03 (0.01–0.07)
Ukraine	7,479	4.3	7,412	99.1	3	40.5	0	0.0	-
Living Condition^e^									
Non-camp	101,307	58.5	98,803	97.5	429	434.2	21	21.3	1.0
Refugee camp	39,050	22.5	38,534	98.7	215	557.9	26	67.5	0.8 (0.6–0.9)

a EDN: Electronic Disease Notification System

b Screening data missing for 12,862 records. 9,075 (70.6%) from the Western Hemisphere and 96% were in the form of paper records and subject to manual data entry errors. Data improvement collection processes have since been implemented

c Positive screening and confirmatory tests. Nyangoma et al. case definition includes documented treatment of syphilis up to 1 year before overseas exam [6]

d Region of origin based on nationality. Categorization defined by DOS:

**Africa (Sub-Sahara):** Angola, Benin, Botswana, Burkina Faso, Burundi, Cabo Verde, Cameroon, Central African Republic, Chad, Comoros, Democratic Republic of the Congo (D.R.C.), Republic of the Congo, Cote d’Ivoire, Djibouti, Equatorial Guinea, Eswatini, Gabon, Gambia, Ghana, Guinea, Guinea-Bissau, Kenya, Lesotho, Liberia, Madagascar, Malawi, Mali, Mauritania, Mauritius, Mozambique, Namibia, Niger, Nigeria, Rwanda, Sao Tome and Principe, Senegal, Seychelles, Sierra Leone, South Africa, South Sudan, Tanzania, Togo, Uganda, Zambia, and Zimbabwe

**East Asia/Pacific:** Australia, Brunei, Cambodia, China, Fiji, Indonesia, Japan, Kiribati, North Korea, South Korea, Laos, Malaysia, Marshall Islands, Federated States of Micronesia, Mongolia, Nauru, New Zealand, Palau, Papua New Guinea, Philippines, Samoa, Singapore, Solomon Islands, Taiwan, Thailand, Timor-Leste, Tonga, Tuvalu, Vanuatu, and Vietnam

**Near East:** Algeria, Bahrain, Egypt, Israel, Jordan, Kuwait, Lebanon, Libya, Morocco, Oman, Palestinian Territories, Qatar, Saudi Arabia, Tunisia, United Arab Emirates, and Yemen

**South & Central Asia:** Bangladesh, India, Kazakhstan, Kyrgyzstan, Maldives, Nepal, Pakistan, Sri Lanka, Tajikistan, Turkmenistan, and Uzbekistan

**Europe & Eurasia:** Albania, Andorra, Armenia, Austria, Azerbaijan, Belarus, Belgium, Bosnia and Herzegovina, Bulgaria, Croatia, Cyprus, Czechia, Denmark, Estonia, Finland, France, Georgia, Germany, Greece, Holy See, Hungary, Iceland, Ireland, Italy, Kosovo, Latvia, Liechten-stein, Lithuania, Luxembourg, Macedonia, Malta, Moldova, Monaco, Montenegro, Netherlands, Norway, Poland, Portugal, Romania, Russia, San Marino, Serbia, Slovakia, Slovenia, Spain, Sweden, Switzerland, Turkey, and United Kingdom

**Western Hemisphere:** Antigua and Barbuda, Argentina, Aruba, Bahamas, Barbados, Belize, Bolivia, Brazil, Canada, Chile, Colombia, Costa Rica, Curacao, Dominica, Dominican Republic, Ecuador, Grenada, Guatemala, Guyana, Haiti, Honduras, Jamaica, Mexico, Nicaragua, Panama, Paraguay, Peru, St. Kitts and Nevis, St. Lucia, St. Maarten, St. Vincent and the Grenadines, Suriname, Trinidad and Tobago, Uruguay, and Venezuela

e Living condition based on U.S. Department of State categorizations of different locations and whether they were non-camp, or urban settings, refugee camps

Unknown/Missing: 32,886 (19%) of total EDN records, 23,044 (14%) of total screened, 53 (7.6%) of seropositive for syphilis, and 6 (11%) of infectious syphilis

**Table 2:** Syphilis screening, staging, and treatment characteristics of refugees ≥ 15 years old who arrived in the United States from January 1, 2015-December 2018

	Syphilis Test				Infectious (Primary and Secondary Cases)	
	Total Screened^a^		Seropositive^b^			
	*N*	%(column)	*n*	%(row)	*n*	Per 100,000
Total	160,381	100	697	0.4	53	33.0
Screening Test^f^						
RPR	95,175	59.3	844	0.9	45	-
VDRL	63,629	39.7	933	1.5	8	-
Treponemal	520	0.3	20	3.8	0	-
Other/Unknown	1,057	0.7	99	9.4	0	-
Confirmatory Test^g^						
TP-HA	1,267	0.8	418	33.0	37	-
FTA-A	91	0.1	6	6.6	0	-
TP-PA	9	0.0	2	22.2	1	-
CIA	1	0.0	1	100.0	0	-
Non-Treponemal	55	0.0	17	30.9	0	-
Other/Unknown	473	0.3	253	53.5	15	-
Syphilis Staging^h^						
Primary	-	-	48	-	-	-
Secondary	-	-	5	-	-	-
Early Latent	-	-	20	-	-	-
Late Latent	-	-	397	-	-	-
Tertiary	-	-	2	-	-	-
Other/Not Stated^i^	-	-	225	-	-	-
Treated						
Yes	-	-	693	-	53	-
No			0	-	0	-
Other^j^	-	-	4	-	0	-
Disease treatment type^k^						
Benzathine penicillin	-	-	622	-	51	-
Doxycycline	-	-	39	-	0	-
Other treatment	-	-	36	-	2	-

a Screening data missing for 12,862 records

b Positive screening and confirmatory tests recorded in EDN. Nyangoma et al. case definition includes documented treatment of syphilis up to 1 year before overseas exam

f Screening testing:

RPR: Rapid Plasma Reagin

VDRL: Venereal Disease Research Laboratory

Treponemal: Confirmatory treponemal tests that were used for screening

Other/Unknown: 91 were SD Bioline, an HIV/Syphilis Duo tests, 29 were TRUST (Toluidine Red Unheated Serum Test), 173 were a Nucleic acid amplification test (NAATs), 2 were a GeneXpert CT/NG assay, and 762 were missing screening test name but did have screening indicated on exam

g Confirmatory testing:

TPHA: *Treponema pallidum* hemagglutination test

FTA-ABS: Fluorescent treponemal antibody absorbed test

TP-PA: *Trepnema pallidum* passive particle agglutination assay

CIA: Chemiluminescence immunoassays

Non-Treponemal: Screening non-treponemal tests that were used as confirmatory screening

Other/Unknown: 124 were SD Bioline, 349 were missing confirmatory test name in exam notes

h Syphilis staging include three infectious stages (primary, secondary, early, and latent) and two non-infectious stages (late latent and tertiary)

i Other/Not Stated: 1 case was non-infectious neurosyphilis and 224 were missing staging data in EDN. 204 of those with missing staging data had previously been treated for syphilis within the past year with no staging data being documented in the exam notes, and 20 lacked any information regarding staging or previous syphilis infection in the exam notes

j 4 missing treatment data were comprised of 3 determined to be false reactive and 1 indicated as missing treatment data but was listed as non-infectious in exam notes

k Syphilis treatment includes benzathine penicillin G (BPG) as the preferred regimen or other alternatives regimens. Other treatment name not specified

## Data Availability

The datasets generated and/or analyzed during the study are not publicly available for the protection of the refugees.
